# High expression of PI4K2A predicted poor prognosis of colon adenocarcinoma (COAD) and correlated with immunity

**DOI:** 10.1002/cam4.4895

**Published:** 2022-05-30

**Authors:** Xinkun Huang, Yang Cao, Peng Bao, Bingye Zhu, Zhouyang Cheng

**Affiliations:** ^1^ Department of General Surgery Affiliated Hospital of Nantong Nantong Jiangsu Province China; ^2^ Department of Operation Affiliated Hospital of Nantong Nantong Jiangsu Province China; ^3^ Department of Critical Care Medicine Affiliated Hospital of Nantong University Nantong Jiangsu Province China; ^4^ Department of Urology Affiliated Nantong Hospital of Shanghai University/The Sixth People's Hospital of Nantong Nantong Jiangsu Province China

**Keywords:** biomarker, colon adenocarcinoma, immunity, PI4K2A, prognosis

## Abstract

**Background:**

PI4K2A has been found to have a tumor‐promoting role in various solid tumors and be involved in various biological procedures. In this article, we aim to investigate the prognostic values of PI4K2A and provide new insights in colon adenocarcinoma (COAD).

**Methods:**

The Cancer Genome Atlas (TCGA) database, Human Protein Atlas online database, and UALCAN database were used to analyze the expression of PI4K2A in COAD and the survival of patients. Univariate and multifactorial Cox regression analyses were used to assess the prognosis of PI4K2A on COAD. GSEA was used to explore PI4K2A‐related signaling pathways. In addition, the effect of PI4K2A on immune checkpoint inhibitors (ICIs) treatment was investigated by constructing a TIDE model and predicting the association between PI4K2A and anticancer drug sensitivity through the CellMiner database.

**Results:**

In the TCGA database, PI4K2A was highly expressed in COAD and the similar results were verified by qRT‐PCR. Survival analysis, utilizing Kaplan–Meier curves, revealed that COAD patients with high PI4K2A expression had a worse prognosis. In addition, PI4K2A expression was discovered to have been associated with T‐stage, N‐stage, and pathological stage by logistic analysis. Next, we utilized univariate and multifactorial Cox regression analyses to identify PI4K2A as an independent predictor. Additionally, GSEA analysis indicates that PI4K2A is enriched in MAPK signaling pathway, Toll‐like receptor signaling pathway, etc. In COAD, PI4K2A was remarkably associated with the tumor immune microenvironment. In addition, by constructing a TIDE model, we discovered that COAD patients in the PI4K2A low‐expression cohort were better treated with ICI. Finally, analysis of the CellMiner database predicted that PI4K2A was adversely correlated with the sensitivity of various anticancer drugs.

**Conclusions:**

Our study suggests that PI4K2A may be a potential predictor of poor prognosis in COAD and a potential biomarker for early diagnosis, prognosis, and treatment.

## Graphical abstract

PI4K2A is a potent potential biomarker for early diagnosis, prognosis, and treatment of COAD. In addition, PI4K2A is an independent prognostic element of COAD and is closely interrelated with immunotherapy. Moreover, many anti‐cancer drug sensitivities were negatively correlated with PI4K2A expression, providing some new ideas for the treatment of COAD. However, our findings need to be further validated by in vivo and in vitro assays.

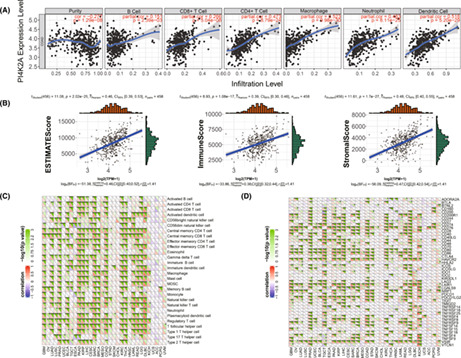

## 1 INTRODUCTION

Worldwide, colon adenocarcinoma (COAD) is a widespread prevalent neoplasm with a high morbidity and mortality level.^1^ In 2020, approximately 1.14 million people were diagnosed with COAD and 570,000 people died from this disease worldwidely.^2^ In China, with the change of lifestyle and diet, more and more people are diagnosed with COAD every year. Not only the mortality rate of COAD is on the rise, but also the trend of COAD patients is younger.^3^ Many researchers have reported that major risk factors for COAD include environmental factors, genetic factors, heavy alcohol consumption, smoking, consumption of red or processed meats, and physical activity, among others.^4^, ^5^ Surgical resection is still the most important treatment for COAD, followed by chemotherapy and radiotherapy. Patients with advanced COAD usually require combination chemotherapy, including cytotoxic agents (5‐fluorouracil, oxaliplatin, and capecitabine) and biologic agents (bevacizumab, panitumumab, and cetuximab).^6^ In spite of continuous research into immunotherapy and targeted therapies, the survival rate for patients with progressive COAD remains low. Only approximately 10% of patients with distant metastases have a 5‐year survival rate.^7^ Nevertheless, the mechanisms of COAD development remained obscure. Therefore, exploring key molecules that influence COAD growth and metastasis and identifying biomarkers for early diagnosis, prognosis, and treatment are the focus of current research.

PI4K family II includes PI4KIIA and PI4KIIB.^8^ Among them, PI4KIIA, located at chromosome 10 q24.2, is the most abundant PI4K protein in mammals, mainly present in trans‐Golgi network and endonucleosomes,^9^, ^10^ and involved in targeting trans‐Golgi network and intracellular transport.^11^, ^12^, ^13^ Previous researchers have shown that PI4KIIA can regulate HIF‐1alpha through the HER‐2/PI3K and ERK cascades, thus participating in tumorigenesis and progression.^14^ Additionally, other research has discovered that inhibition of PI4K2A along with EGFR inhibition better inhibits EGFR‐dependent tumor growth.^15^ Moreover, PI4K2A has been shown to be associated with additional signaling pathways, such as participation in the WNT pathway^16^, ^17^ or influencing the activation of AKT.^18^ In recent years, some investigators have found that abnormal upregulation of PI4K2A expression can promote the development of breast tumors and is a potential poor prognostic factor.^19^, ^20^, ^21^

Unfortunately, the mechanism of action of PI4K2A in COAD has not been explored. Therefore, we utilized bioinformatics to investigate the biological function of PI4K2A in COAD and found that PI4K2A expression was upregulated in COAD and was linked to worse outcomes. Furthermore, utilizing univariate and multifactorial Cox regression analysis, PI4K2A was identified as an independently predictive element for COAD. Then, the relevant pathways regulated by PI4K2A were confirmed by GSEA. Moreover, we also performed immune correlation analysis of PI4K2A expression levels in COAD, and proposed the relationship between PI4K2A and immunotherapy for the first time by constructing a TIDE model. Finally, we predicted the connection between PI4K2A and anticancer drug susceptibilities by utilizing the CellMiner database to provide better new therapeutic strategies for COAD.

## 2 MATERIALS AND METHODS

### 2.1 Collecting and processes data in TCGA database

In The Cancer Genome Atlas dataset (TCGA; https://tcga‐data.nci.nih.gov/tcga/), expression data and relevant clinical messages were collected for 39 non‐cancerous colon tissues and 398 COAD tissues. All data were log2‐transformed and normalized for analysis using R software.^22^ Differentially expressed genes (DEGs) were identified during the analyses using limma, and genes with adjusted *p*‐values (FDR) < 0.05 and |log2FC| > 1 were selected for the analyses.

### 2.2 Quantitative real‐time PCR (qRT‐PCR) analysis

Utilizing TRIzol reagent (Invitrogen), we abstracted total RNA from COAD and paraneoplastic tissues and detected the expression of PI4K2A with qRT‐PCR.^23^ Primers were synthesized by Sangon and included PI4K2A (Forward: 5’‐GAGGATCCTGAGTTCGAGG‐3′; Reverse: 5’‐GTCCTTGACGAAGTAGCTTCC‐3′) and GAPDH (Forward: 5’‐AGAAGGCTGGGGCTCATTTG‐3′; Reverse: 5′‐ AGGGGCCATCCACAGTCTTC‐3′). The Affiliated Hospital of Nantong University collected eight paired COAD tissues and adjacent non‐cancerous colon tissues from COAD patients. Ethical approval and well‐informed documented consent were granted to all subjects by the Ethics Committee of Nantong University Hospital (NO.2022‐L130).

### 2.3 Analysis of PI4K2A in HPA database and construction of PPI network

PI4K2A protein expression in COAD was verified by immunohistochemical staining of the Human Protein Atlas (HPA, http://www.proteinatlas.org/) online database. The PPI network was also structured through the STRING (https://string‐db.org/) database to explore genes with potential functional interactions with PI4K2A.

### 2.4 Analysis of PI4K2A in UALCAN database

In the current research, the expression of PI4K2A total protein in COAD tissues or normal tissues was explored by the UALCAN database, and the relationship between PI4K2A total protein and histological or pathological stages of COAD was investigated. In addition, we found that PI4K2A was phosphorylated at the S47 site and explored the relationship between PI4K2A phosphorylated at the S47 site and the histological or pathological stage of COAD.^24^ All of the above analyses were performed by using CPTAC.^25^

PI4K2A ROC curve analysis and logistic regression analysis.

Performing time‐dependent ROC analysis utilizing the “timeROC” package of R software, we assessed the specificity and sensitivity of PI4K2A to COAD. In addition, logistic regression analysis was performed to examine the link between PI4K2A expression and clinicopathological parameters in patients with COAD.^26^

### 2.5 Univariate and multifactor Cox risk regression analysis of PI4K2A

Univariate and multivariate Cox regression analyses were done to confirm whether PI4K2A and clinicopathological parameters were independent factors relating to COAD.

### 2.6 GSEA analysis of PI4K2A

Gene Set Enrichment Analysis (GSEA) prediction is widely used in many studies, and it is a method used to determine whether marker genes predict statistical differences between two biological states, and its predictive function has been successfully validated.^27^ To explore the crucial biological pathways of PI4K2A, this research utilized GSEA to analyze the apparent difference in viability between the PI4K2A high‐ and low‐expression cohorts, with at least 1000 ranking tests performed per analysis.

### 2.7 PI4K2A alteration analysis

The cBioPortal for Cancer Genomics (http://cbioportal.org) was used to examine the frequency, mutation type, and CNA (copy number alteration) of PI4K2A alterations in all TCGA tumors. In addition, we explored the differences in overall survival (OS), progression‐free survival (PFS), disease‐free survival (DFS), and disease‐specific survival (DSS) in COAD with and without PI4K2A gene alterations by Kaplan–Meier (K‐M) survival curves.^28^, ^29^

### 2.8 Relationship between PI4K2A and MSI, TMB, TNB

We explored the correlation of PI4K2A gene expression with tumor mutational burden(TMB) or microsatellite instability(MSI) or tumor neoantigen burden(TNB) by employing the Spearman's method^30^, ^31^ using the free online platform Sangerbox tool (http://www.sangerbox.com/tool).^32^, ^33^ Then, it is visualized with radar plots using the R package.

### 2.9 The association of PI4K2A gene expression with tumor immunity in COAD

We used the free online platform Sangerbox tool,^32^, ^33^ set *p* < 0.001 as a threshold, and applied the ESTIMATE algorithm to the expression matrix of PI4K2A to calculate the immune fraction, stromal fraction, and ESTIMATES fraction.^34^ Similarly, with a threshold of *p* < 0.001, we also calculated the immune cell infiltration of COAD by the CIBERSORT algorithm.^35^ Furthermore, we analyzed the association between PI4K2A expression and immune checkpoint molecules or immune cell pathways utilizing the “limma” package, visualized using the R software “reshape2” package and the “RColorBrewer” package.

### 2.10 Relationship between PI4K2A expression and TIDE prediction or drug sensitivity

Prediction of PI4K2A gene expression in relation to immune checkpoint inhibitors (ICI) treatment efficacy to be performed with the tumor immune dysfunction and rejection (TIDE).^36^, ^37^ Moreover, the correlation between PI4K2A expression and drug sensitivity was analyzed by the CellMiner database (http://discover.nci.nih.gov/CellMiner/) and visualized using R.^38^

## 3 RESULTS

### 3.1 PI4K2A mRNA expression levels in COAD correlate with prognosis

**FIGURE 1:**
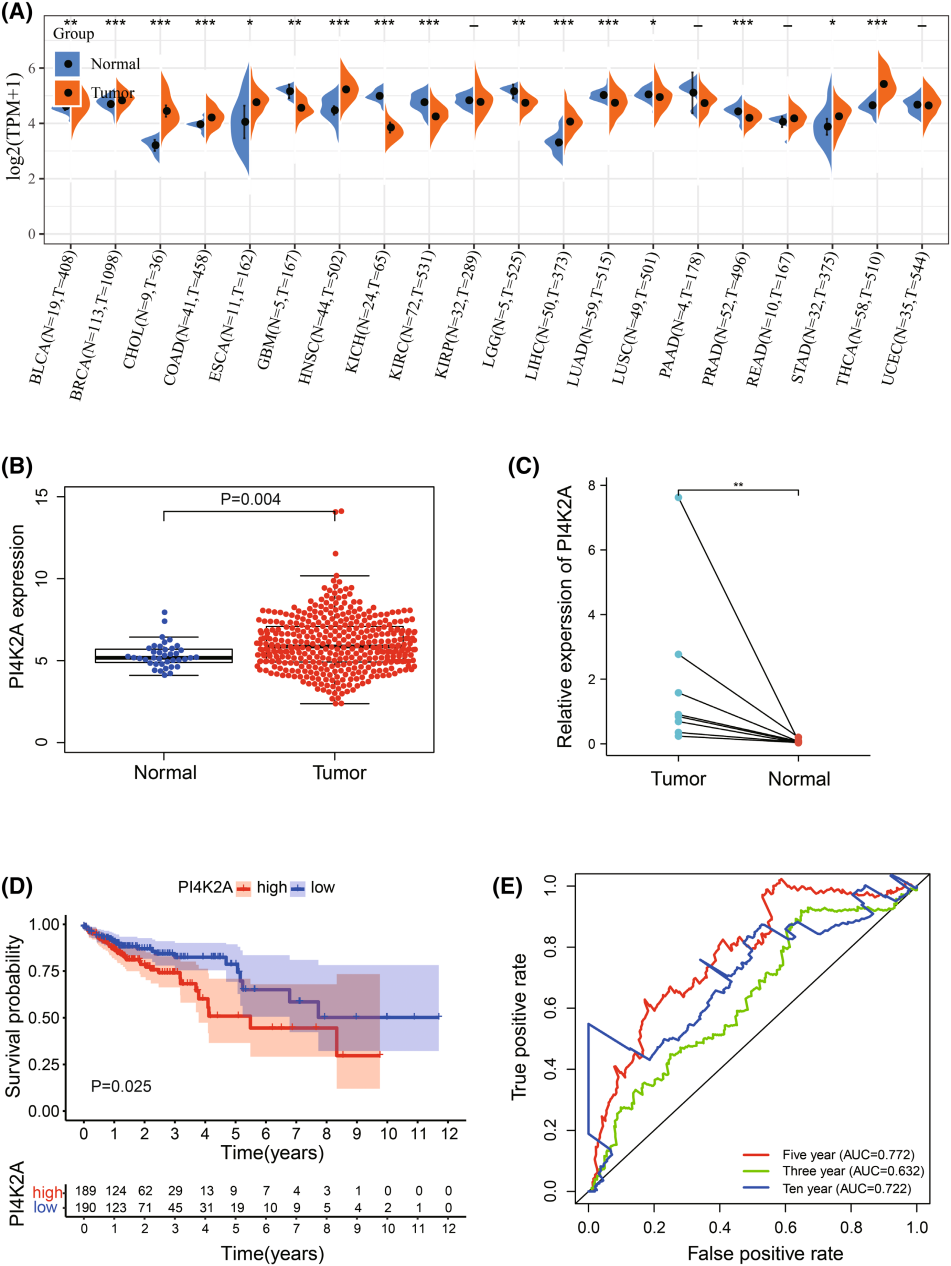
PI4K2A mRNA expression levels in COAD correlate with prognosis. (A) PI4K2A expression in pan‐cancer. (B) Relative expression of PI4K2A in the TCGA database in COAD and non‐cancerous colon tissues (*T* = 398, *N* = 39). (C) PI4K2A expression in eight paired normal and COAD tissues by qRT‐PCR. (D) TCGA database survival curve of PI4K2A expression in COAD. (E) Time‐dependent ROC analysis of PI4K2A expression in COAD in TCGA database with 3, 5, and 10 years

First, we found that PI4K2A expression was upregulated in 458 COAD samples versus 41 non‐cancerous samples in the TCGA database (*p* < 0.01; Figure 1A,B). Besides, we obtained the same results for quantitative real‐time PCR detection of PI4K2A expression according to eight pairs of COAD tissues and paired non‐cancerous tissues (*p* < 0.01; Figure 1C). Then, taking the median expression of PI4K2A in COAD as the threshold, COAD samples were sorted into high‐ and low‐ expression clusters. The K‐M curves revealed that the PI4K2A high‐expression cohort had fewer OS than the PI4K2A low‐expression cohort (*p* < 0.05; Figure 1D). Finally, the time‐dependent ROC indicated AUC values of 0.632, 0.772, and 0.722 for 3, 5, and 10 years, accordingly (Figure 1E). Additionally, it is suggested that PI4K2A has the ability to predict the prognosis of COAD.

### 3.2 Expression of total protein or phosphorylated protein expression levels of PI4K2A in COAD

**FIGURE 2:**
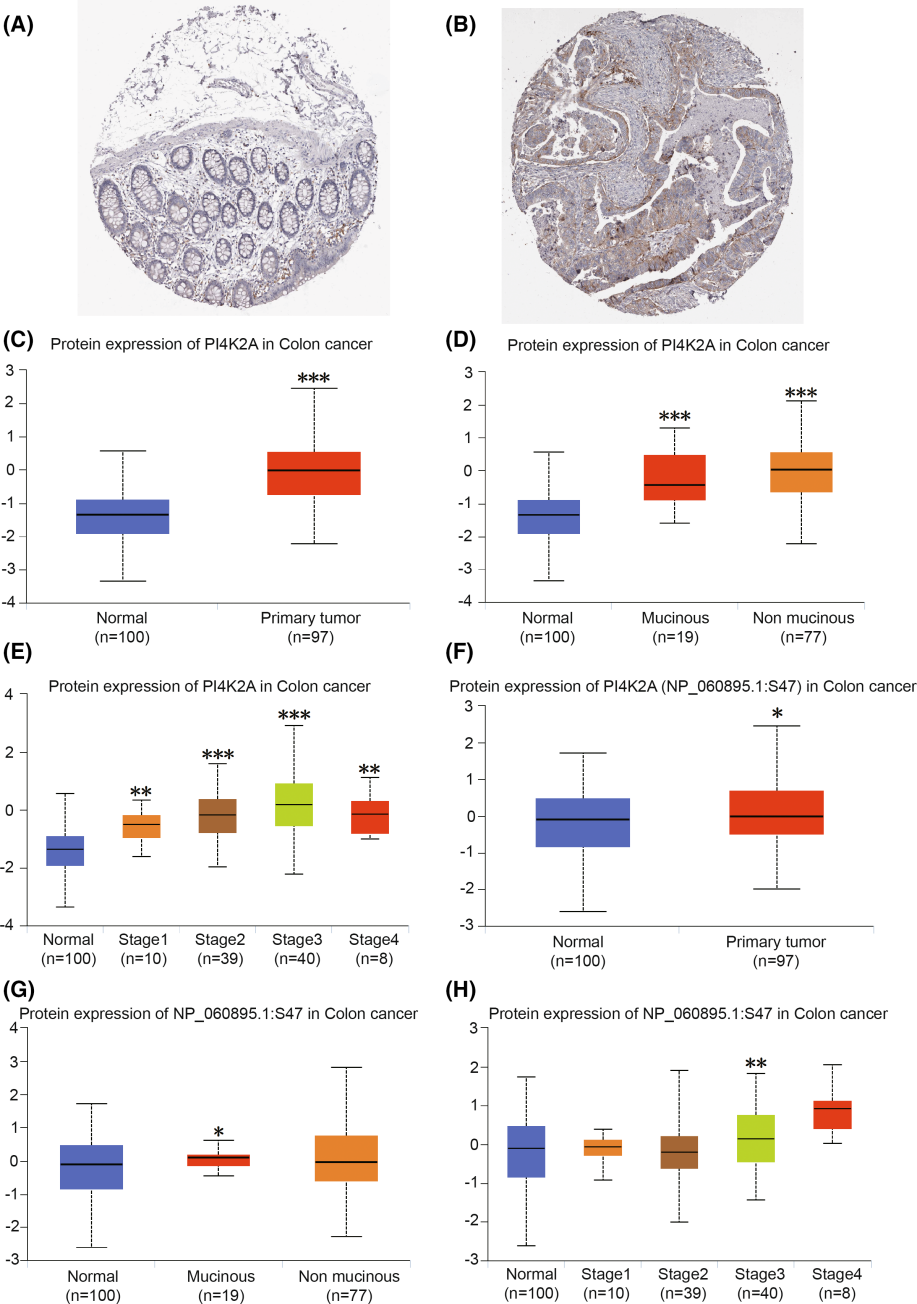
Expression of total protein or phosphorylated protein expression levels of PI4K2A in COAD. (A,B) Immunohistochemical microarray analysis of PI4K2A protein expression in non‐cancerous colorectal tissues or colorectal cancer tissues in the HPA database. Generally, brownish yellow staining in cytoplasm or nucleus is positive for immunohistochemistry and scale bar = 200 μm; (C) PI4K2A protein expression in COAD or normal colonic tissue. (D) PI4K2A protein expression in normal colon tissue or mucinous COAD tissue or non‐mucinous COAD tissue. (E) PI4K2A protein expression in normal colonic tissues and COAD tissues with different pathological stages. (F) Phosphorylated PI4K2A protein expression at the S47 site in COAD or normal colon tissue. (G) Phosphorylated PI4K2A protein expression at the S47 site in normal colon tissue or mucinous COAD tissue or non‐mucinous COAD tissue. (H) PI4K2A protein expression phosphorylated at S47 site in normal colon tissues or in COAD tissues with different pathological stages

PI4K2A expression is aberrantly raised in colorectal cancer tissues in immunohistochemical microarrays in the HPA database (Figure 2A,B). Then, we discovered that PI4K2A was remarkably overexpressed in COAD tissues compared to non‐cancerous colon tissues in the UALCAN database (*p* < 0.001; Figure 2C). And PI4K2A protein expression level correlated with COAD histology and stage (*p* < 0.01; Figure 2D,E). The changes in protein levels were complex, including a series of posttranscriptional modifications such as phosphorylation and ubiquitination. Next, we found that the S47 site of PI4K2A protein was phosphorylated in COAD patients (*p* < 0.05; Figure 2F) and that S47 site phosphorylation in PI4K2A correlated with histological Mucinous classification (*p* < 0.05; Figure 2G) and stage III (*p* < 0.01; Figure 2F). It further demonstrates that PI4K2A expression was aberrantly increasing in COAD tissues.

### 3.3 Relationship between PI4K2A expression and clinicopathological parameters

**FIGURE 3:**
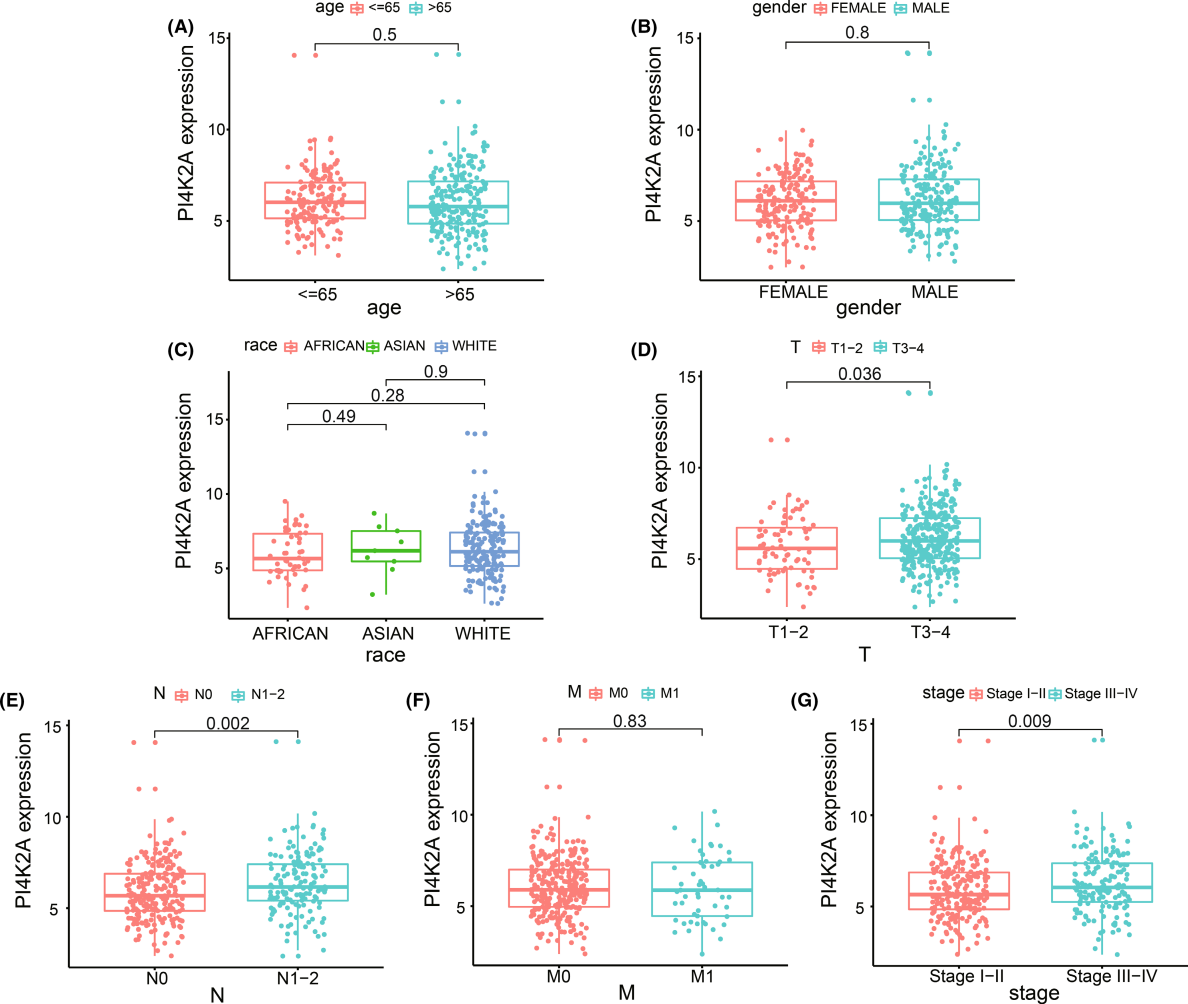
Relationship between PI4K2A expression and clinicopathological parameters. (A) Age. (B) Gender. (C) Race. (D) T. (E) N. (F) M. (G) stage

Subsequently, we investigated the association between PI4K2A expression levels in COAD and the clinicopathological characteristics of COAD patients. The outcomes demonstrated that the PI4K2A expression in COAD has been linked to the T‐stage (*p* = 0.036; Figure 3D), N‐stage (*p* = 0.002; Figure 3E), and pathological stage (*p* = 0.009; Figure 3G). While PI4K2A expression levels were not significantly different in age (Figure 3A), sex (Figure 3B), race (Figure 3C), and M stage (Figure 3F).

### 3.4 Relationship between PI4K2A mutation and prognosis in COAD

**FIGURE 4:**
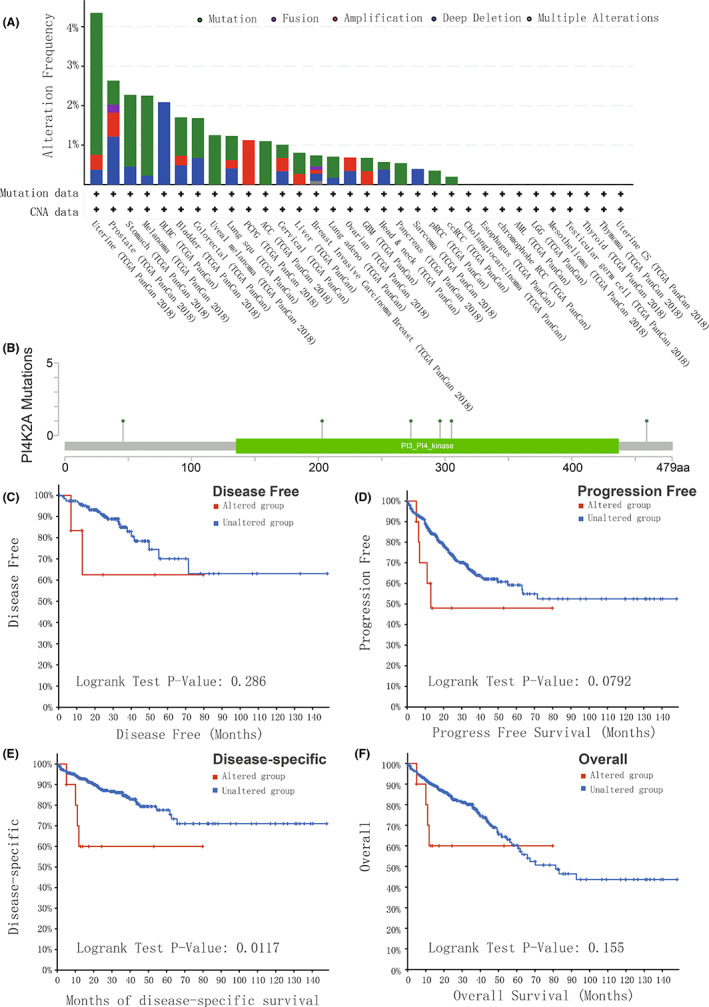
Mutation characteristics of PI4K2A in COAD and its prognosis were analyzed using the cBioPortal database. (A) Frequency of alteration of PI4K2A mutation type in pan‐cancer in the TCGA database. (B) Mutation sites of PI4K2A in COAD in the TCGA database. (C) K‐M survival analysis of PI4K2A mutations on DFS. (D) K‐M survival analysis of PI4K2A mutations on PFS. (E) K‐M survival analysis of PI4K2A mutations on DSS. (F) K‐M survival analysis of PI4K2A mutations on OS

We also investigated the frequency of PI4K2A mutations in cancer utilizing the cBioPortal (TCGA, pan‐cancer Atlas) database, which indicated that the frequency of PI4K2A alterations in colorectal cancer patients was approximately 1.7% (Figure 4A), and its mutation sites are shown in Figure 4B. K‐M analysis indicates that the DSS is worse in patients with PI4K2A gene mutation than in patients without PI4K2A gene mutation (*p* = 0.0117; Figure 4E). However, mutations in PI4K2A in COAD were not statistically correlated with DFS (Figure 4C), PFS (Figure 4D), and OS (Figure 4F).

### 3.5 PI4K2A is an independently predictive element of COAD

**FIGURE 5:**
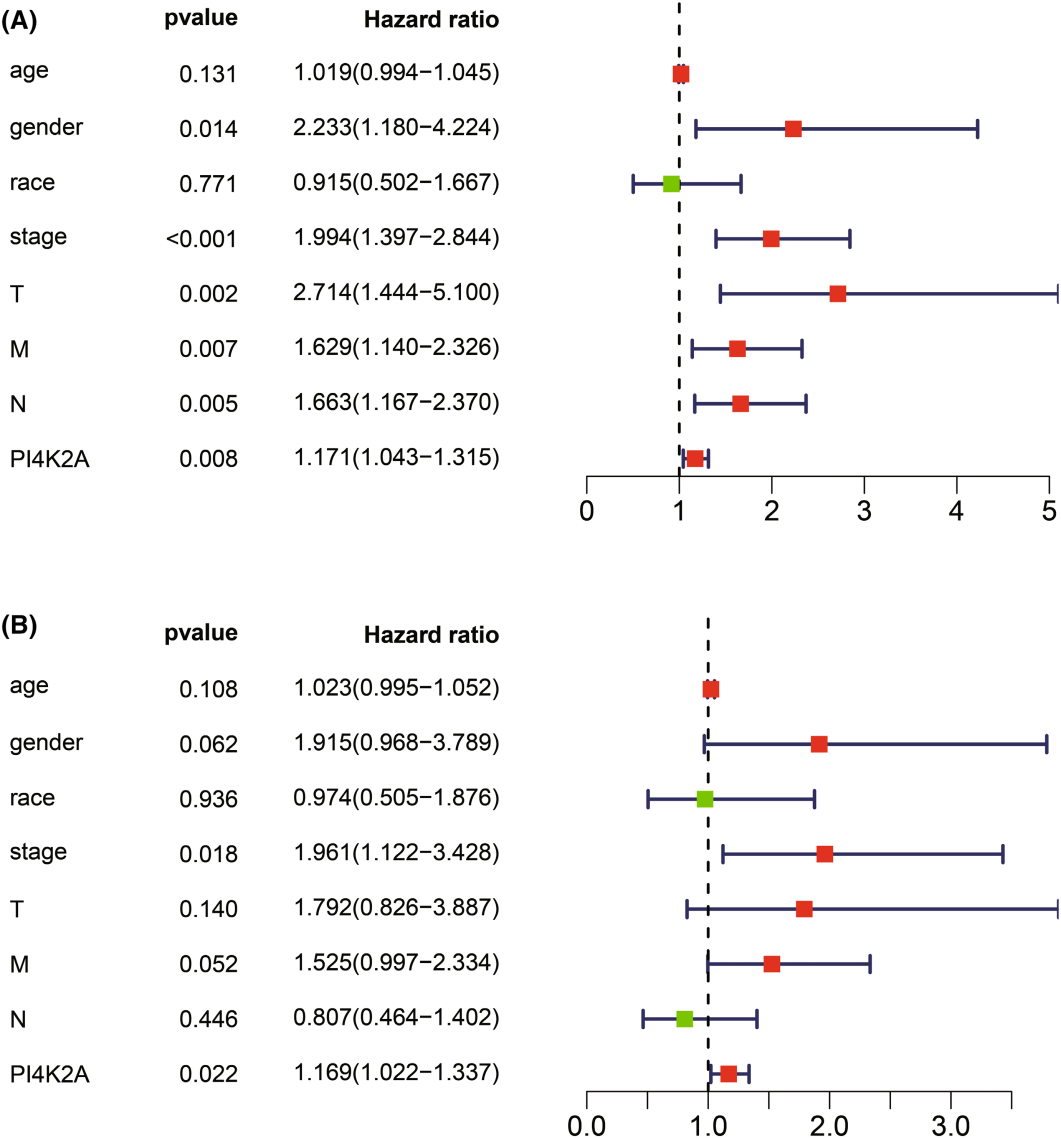
Univariate and multivariate Cox analysis of PI4K2A in COAD. (A) Univariate Cox analysis was utilized to analyze the relationship between PI4K2A expression and clinicopathological variables in COAD in the TCGA database. (B) Multivariate Cox was utilized to analyze the relationship between PI4K2A expression and clinicopathological variables in COAD in the TCGA database

Subsequently, we performed univariate Cox and multivariate Cox regression analyses to further investigated the influences of PI4K2A on the prognosis of COAD. (Table 1). Univariate Cox analysis showed that PI4K2A expression, gender, pathological stage, T‐stage, N‐stage, and M‐stage were significantly associated with overall survival in patients with COAD (*p* < 0.05; Figure 5A). As illustrated in Figure 5B, multivariate Cox analysis reveals that PI4K2A is an independent predictor of OS in COAD. Hence, our results suggest that P4K2A expression is an independent prognostic factor in COAD.

**TABLE 1:** Univariate and multivariate Cox hazard regression analyses of PI4K2A in TCGA‐COAD dataset

Characteristics	Univariate analysis				Multivariate analysis			
	HR	HR.95L	HR.95H	*p* value	HR	HR.95L	HR.95H	*p* value
Age	1.019482	0.994269	1.045334	0.131014	1.023303	0.994947	1.052467	0.10813
Gender	2.23311	1.180471	4.224399	**0.013509**	1.914625	0.967572	3.788648	0.06214
Race	0.914905	0.502185	1.666818	0.771367	0.973673	0.505331	1.876077	0.936454
Stage	1.993568	1.397495	2.843882	**0.000141**	1.961339	1.122256	3.427785	**0.018036**
T	2.713683	1.444052	5.099591	**0.001925**	1.792169	0.826338	3.886869	0.139664
M	1.628803	1.14046	2.326253	**0.007303**	1.525185	0.996504	2.33435	0.051915
N	1.663132	1.167274	2.369631	**0.004859**	0.806779	0.464181	1.402239	0.446492
PI4K2A	1.171058	1.042562	1.315391	**0.007748**	1.168957	1.022413	1.336504	**0.022354**

The bold values represent *p*‐values < 0.05.

### 3.6 GSEA analysis of PI4K2A in COAD

**FIGURE 6:**
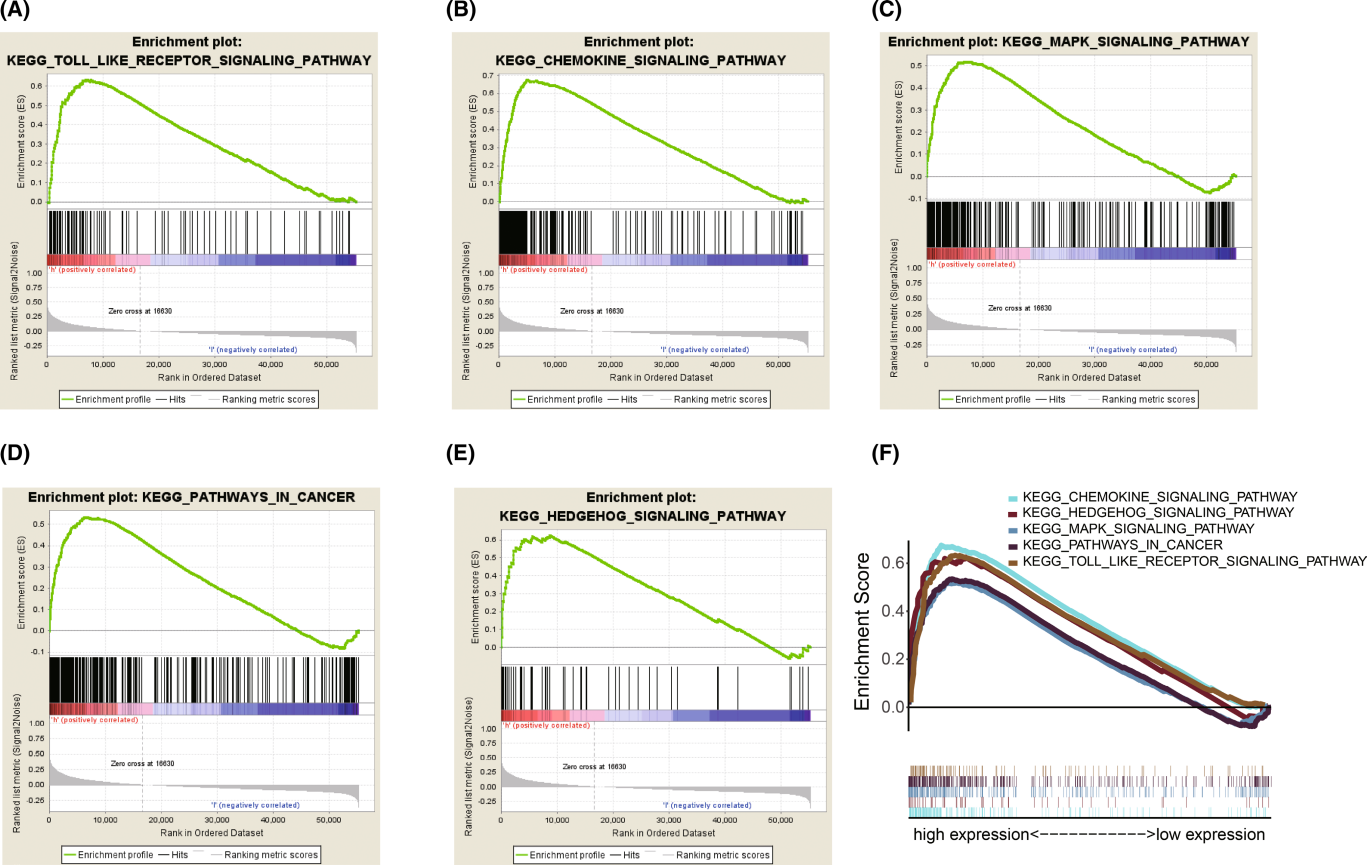
PI4K2A‐related signaling pathways identified by gsea. (A) Toll‐like receptor signaling pathway. (B) Chemokine signaling pathway. (C) MAPK signaling pathway. (D) Pathways in cancer. (E) Hedgehog signaling pathway. (F) The five most significantly enriched PI4K2A‐related pathways

Although we found that PI4K2A may be an independent predictor of COAD, the mechanism of how PI4K2A contributes to the development of COAD is unclear. Therefore, we investigated the mechanism and signaling pathway of PI4K2A regulation of COAD utilizing GSEA analysis. We categorized the five clearest enriched signaling pathways by the Normalized Enrichment Score (NES) and FDR q‐val (FDR <0.01), which were Chemokine signaling pathway, Hedgehog signaling pathway, MAPK signaling pathway, Pathways in cancer, and Toll‐like receptor signaling pathway (Table 2; Figure 6A–F), providing clues to the pathogenesis of COAD.

**TABLE 2:** Gene set enrichment analysis results

MSigDB collection	Gene set name	NES	NOM *p*‐val	FDR *q*‐val
c2.cp.kegg.v7.1.symbols.gmt	KEGG_CHEMOKINE_SIGNALING_PATHWAY	2.419	<0.001	<0.001
	KEGG_HEDGEHOG_SIGNALING_PATHWAY	2.247	<0.001	<0.001
	KEGG_MAPK_SIGNALING_PATHWAY	2.198	<0.001	<0.001
	KEGG_PATHWAYS_IN_CANCER	2.228	0.002	<0.001
	KEGG_TOLL_LIKE_RECEPTOR_SIGNALING_PATHWAY	2.368	<0.001	<0.001

### 3.7 Construction of PI4K2A PPI network in COAD and association with MSI, TMB, and neoantigens

**FIGURE 7:**
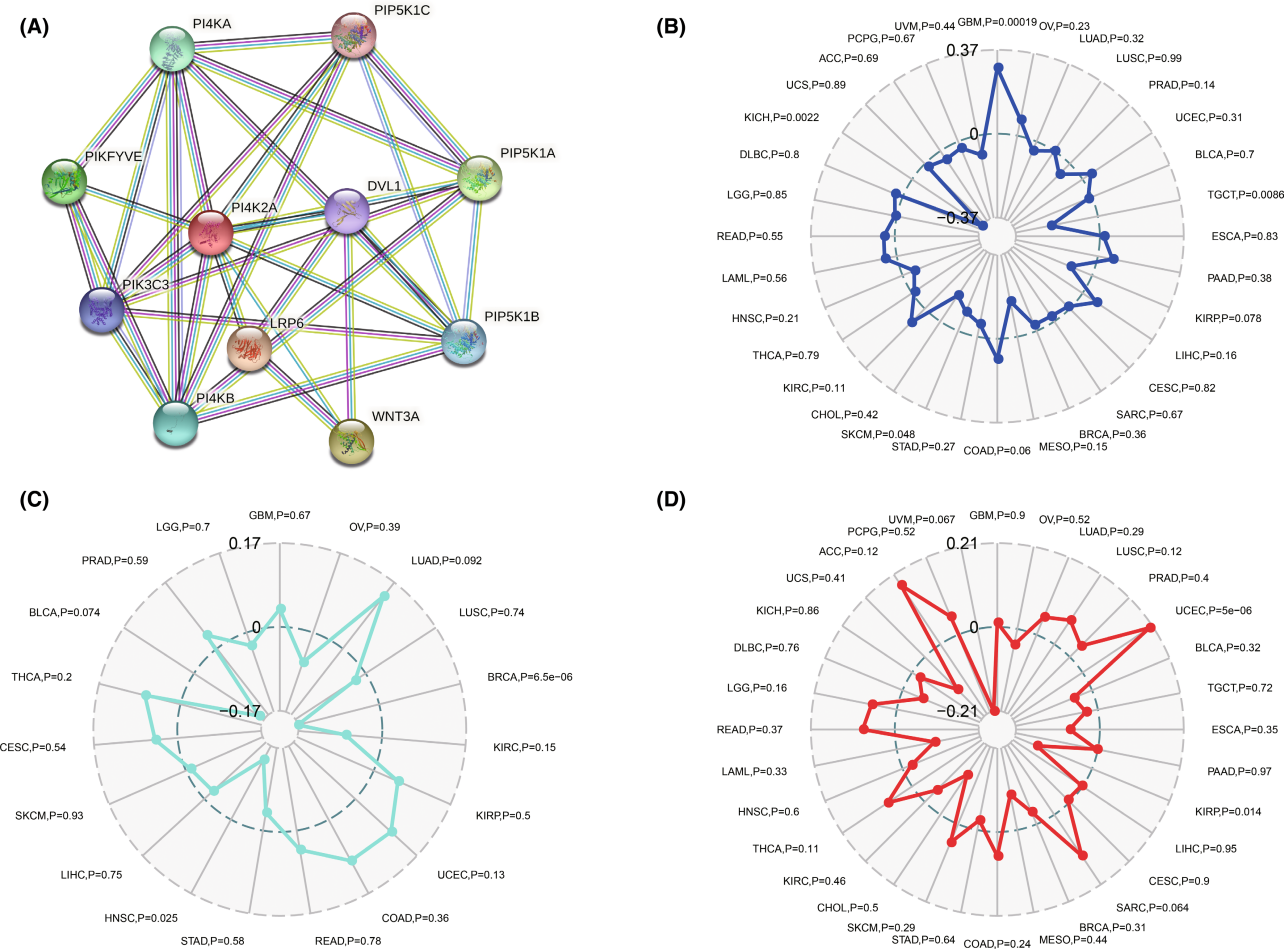
PPI network of PI4K2A and association of PI4K2A with MSI, TNB, and TMB in COAD. (A) PPI network associated with PI4K2A. (B) Association of PI4K2A with MSI. (C) Association of PI4K2A with TNB. (D) Association of PI4K2A with TMB

Subsequently, we built a PPI network by leveraging the STRING database to investigate the underlying interacting proteins of PI4K2A, as shown in Figure 7A, 10 genes (PI4KA, PIP5KIC, PIKFYVE, PIP5K1A, PIP5K1B, DVL1, WNT3A, LRP6, PI4KB, and PIK3C3) were significantly correlated with PI4K2A function. In addition, we further investigated whether PI4K2A was associated with MSI, neoantigen, or TMB by using COAD samples from the TCGA database. The outcomes indicated no statistically meaningful relationship between PI4K2A and MSI (*p* = 0.6), neoantigen (*p* = 0.36), and TMB (*p* = 0.24) (Figure 7B–D).

### 3.8 Immunological features associated with PI4K2A in COAD

**FIGURE 8:**
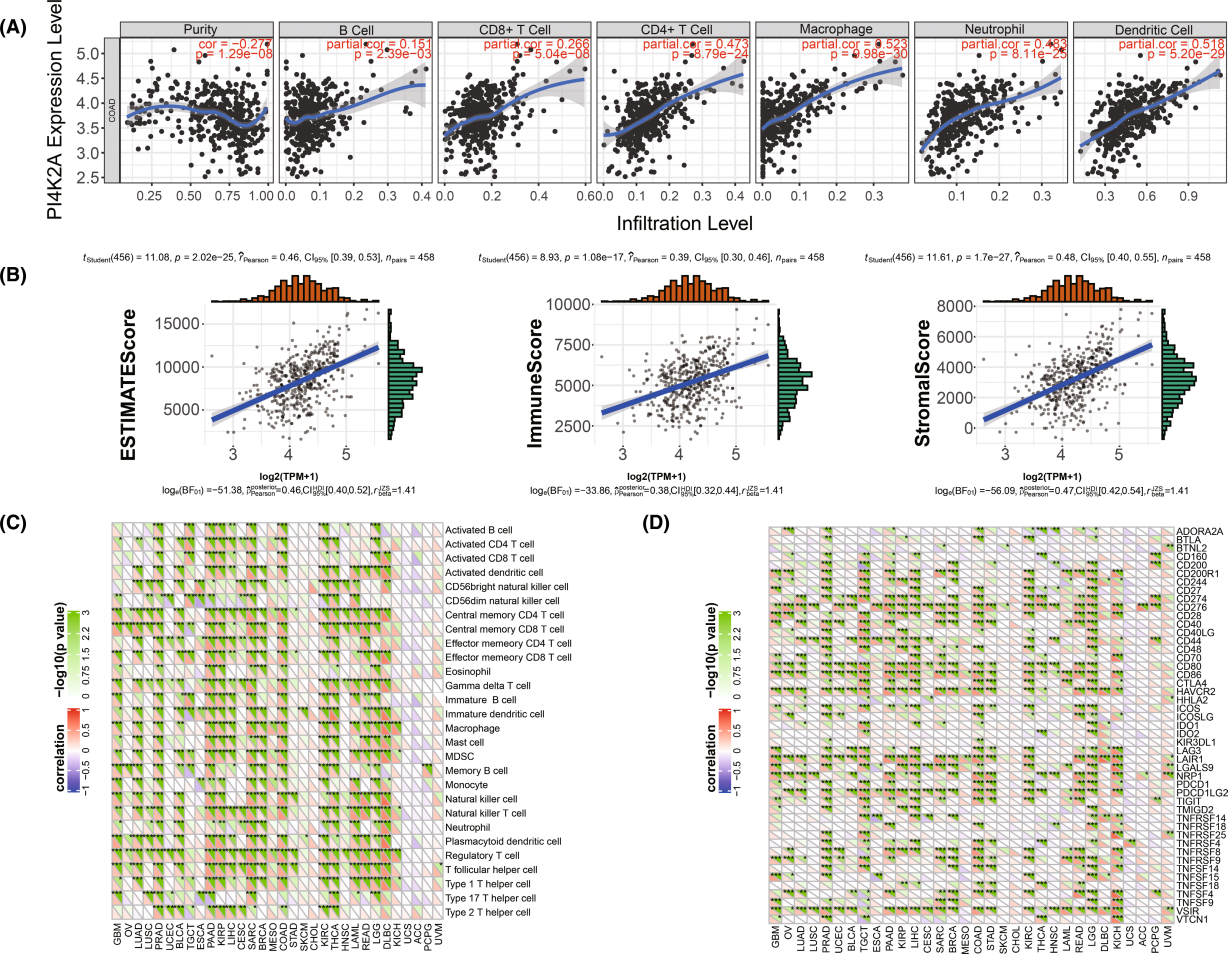
Immune features associated with PI4K2A in COAD. (A) Relationship between PI4K2A expression and immune cell infiltration in COAD. (B) Relationship between PI4K2A expression and tumor immune microenvironment in COAD. (C) Relationship between PI4K2A expression and immune checkpoint gene expression in COAD. (D) Association of PI4K2A with immune cellular pathways in COAD

We investigated the relationship of PI4K2A with immune infiltration and tumor microenvironment in COAD using TIMER and ESTIMATE web tools. Then, PI4K2A was linked to the level of six immune cell infiltrations by the TIMER network tool and revealed to be connected to the level of immune cell infiltration (*p* < 0.001; Figure 8A). We used the “ESTIMATE” package of R software to investigate the immune and stromal scores of COAD samples and discovered that PI4K2A expression was positively correlated with immune and stromal scores. Next, the immune score and the stromal score were combined to obtain an estimated score, and PI4K2A expression was also revealed to be positively correlated with the estimated score (*p* < 0.001; Figure 8B). Associations between PI4K2A and the immune microenvironment of COAD tissues in the TCGA database were further investigated to explore potential targets for immunotherapy of COAD. Correlation analysis showed that PI4K2A was significantly correlated with the expression of immune checkpoint genes such as PDCD1, CD274, CD80, and CD86 (*p* < 0.001; Figure 8C). Additionally, we investigated the link between PI4K2A expression levels and 28 immune cell infiltration scores in COAD, and PI4K2A was remarkably relevant to immune cell infiltration (including activated‐B cells, macrophages, natural killer cells, etc.) (*p* < 0.001; Figure 8D).

### 3.9 Estimation of cancer immunotherapy response using PI4K2A expression in TCGA

**FIGURE 9:**
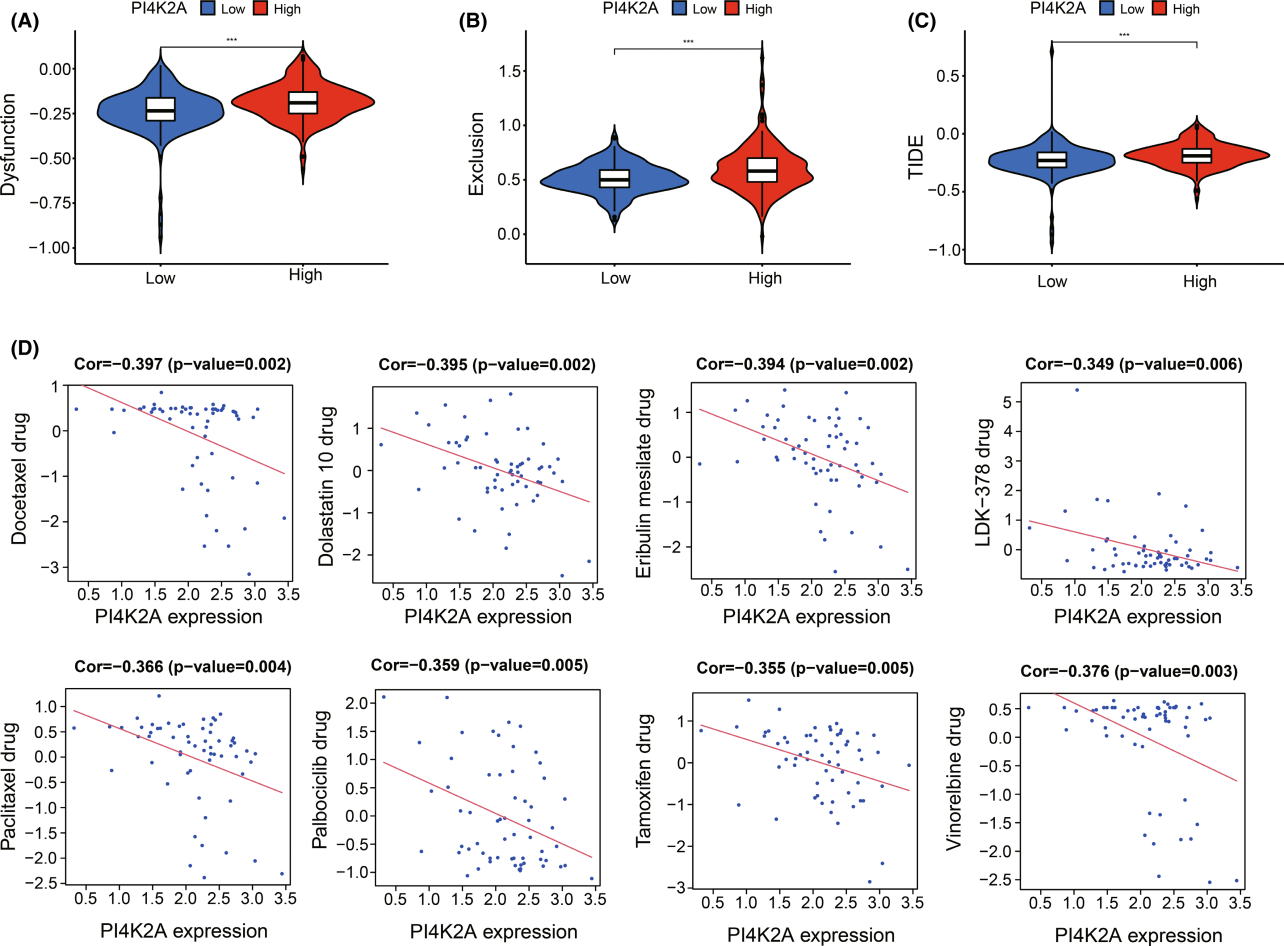
TIDE model and the relationship with anticancer drug sensitivity. (A) Relationship between PI4K2A expression and immune dysfunction. (B) Relationship between PI4K2A expression and immune exclusion. (C) Relationship between PI4K2A expression and TIDE. (D) Relationship between PI4K2A expression and sensitivity to multiple anticancer drugs

Next, we utilized TIDE to forecast the effect of PI4K2A expression on immunotherapy in COAD. A stronger TIDE prediction score indicates a greater likelihood of immune evasion, which means that patients are unlikely to gain advantage from ICI treatment.^39^ We discovered that the TIDE score was less in the PI4K2A low‐expression cohort versus the PI4K2A high‐expression cohort, implying that ICI treatment was more beneficial for patients with lower PI4K2A expression (*p* < 0.001; Figure 9A–C).

### 3.10 Relevance of PI4K2A expression to anticancer drug sensitivity

The association relationship between PI4K2A and antineoplastic agents susceptibility was investigated by CellMiner database, and eight antineoplastic agents with remarkable relevance to PI4K2A expression were filtered out. As shown in Figure 9D, we found a significant negative correlation of PI4K2A with Docetaxel, Dolastatin 10, Eribulin mesilate, LDK‐378, Paclitaxel, Palbociclib, Tamoxifen, and Vinorelbine (*p* < 0.01).

## 4 DISCUSSION

There have been great developments in the screening, diagnosis, and treatment of COAD in the past several years, but the prognosis remains unsatisfactory. Moreover, COAD continues to have a high fatality rate and a very poor 5‐year survivorship. Fortunately, the improved prognosis of COAD patients has made personalized treatment possible, with the development of prepared prognostic markers and biological therapeutic targets. In recently, many researches have reported that PI4K2A is closely related to tumor development,^14^, ^15^, ^19^ but very few have focused on the connection between PI4K2A and the prognosis of COAD prediction. Therefore, our current study focuses on the prognostic worth and therapeutic possibility of PI4K2A in COAD.

We discovered an elevated level of PI4K2A expression in COAD versus non‐cancerous tissues in the TCGA database. In addition, we also performed q‐PCR on the express of PI4K2A in eight couples of COAD tissues and non‐cancerous tissues collected from our hospital and obtained consistent results. Then, K‐M curve analysis demonstrates worse OS in patients with high PI4K2A expression. The AUCs were 0.632, 0.772, and 0.722 at 3, 5, and 10 years, respectively, based on the time‐dependent ROC analysis. Therefore, we considered PI4K2A as a poor prognostic factor for COAD. Then, PI4K2A protein expression levels were analyzed using immunohistochemical microarrays from the HPA database and the CPTAC database. The outcomes displayed that the expression of PI4K2A protein was highly increased in COAD tissues versus non‐cancerous colon tissue. Studies have demonstrated that posttranslational modifications (PTMs) regulate a variety of cellular functions, and phosphorylation is one of the most studied PTMs, involved in such processes as cell growth, differentiation, apoptosis, and cell signaling.^15^, ^40^, ^41^ Interestingly, we discovered that PI4K2A protein was more phosphorylated in COAD tissues than in non‐cancerous tissues and that phosphorylation of this protein was related to a higher T‐stage. Meanwhile, PI4K2A expression was remarkably linked to T‐stage, N‐stage, and pathological stage. In addition, we discovered that PI4K2A would be mutated in COAD, mainly through Mutation and Deep Deletion. K‐M survival analysis revealed that mutations in PI4K2A and worse DSS were associated. Univariate and multivariate COX regression analyses were performed utilizing TCGA database data and clinical data to explore the potential effect of PI4K2A on COAD, and PI4K2A was identified as an independent factor for prognosis.

To investigate as to how PI4K2A is engaged in the development of COAD, we conducted GSEA to find that PI4K2A may be involved in chemokine signaling pathway, hedgehog signaling pathway, MAPK signaling pathway, pathway in cancer, and Toll‐like receptor signaling pathway. Interestingly, Chemokine signaling pathway was also reported as being involvement in immune resistance mechanisms in gastrointestinal malignancies,^42^ while Hedgehog signaling pathway was associated with macrophage polarization and inhibition of CD8+ T cell recruiting in cancer.^43^ Furthermore, MAPK signaling pathway has a pivotal role in immunotherapy,^44^, ^45^ and Toll‐like receptor signaling pathway has been reported to affect the recruitment of monocyte‐derived dendritic cells and the production of Treg.^46^ Among them, the complex crosstalk between COAD cells and the tumor immune microenvironment plays an essential part in regulating COAD behavior.^47^, ^48^ Therefore, we not only constructed a PPI network of PI4K2A through which to investigate underlying protein–protein interactions, but also conducted several correlative researches to discover the possibility of PI4K2A in the immunotherapy of COAD. Although no clear correlation was found between PI4K2A and immunotherapy‐related features such as neoantigens, MSI, TMB, and tumor microenvironment, immune cell correlation analyses identified that PI4K2A expression levels were corresponded to immune cell infiltration such as CD4+ infiltration, macrophage infiltration, neutrophil infiltration, and dendritic cell infiltration were closely correlated. Additionally, we discovered that the expression level of PI4K2A in COAD was obviously linked to the expression of immune checkpoint genes such as PDCD1, CD274, CD80, and CD86. It is well documented that the high expression of PDCD1 (PD‐1, CD279) and PD‐L1 (CD274) is closely related to bad prognosis in certain human cancers.^30^, ^49^ Increasingly, it was also demonstrated that cancers with higher PD‐L1 expression affect regulatory‐T cells in the cancer microenvironment.^50^ The TIDE Predictive Index score is a computing framework that was developed for predicting immunotherapy and has been used extensively in many studies in recent years where its forecasting function has been successfully proven.^37^ In what we studied, the PI4K2A low‐expression cohort responded much better to ICI treatment, as predicted by TIDE. According to these outcomes, we inferred that PI4K2A may be a reliable immune biomarker in COAD treatment.

Finally, we determined the connection between PI4K2A expression levels and anticancer drug sensitivity availing of the CellMiner database. Based on the results, PI4K2A expression correlated negatively with the sensitivity to most anticancer drugs, including Docetaxel, Paclitaxel, and Tamoxifen. In previous studies, docetaxel was found to have potential in targeted therapy for COAD.^51^, ^52^ And PI3K/mTOR Dual Inhibitor BEZ235 and Nano‐Emulsioned Paclitaxel were reported to be used to treat drug‐resistant COAD.^53^ Therefore, in our prediction, a possible new idea is provided for COAD patients with high PI4K2A expression can be treated with docetaxel or paclitaxel in combination with conventional therapy. It can be observed that the upregulation of PI4K2A expression may have influenced the outcome of chemotherapy for COAD. It may lead to some bias in our *result* owing to the few amounts of non‐cancerous colon tissue samples in the TGGA database (N = 39). And whether PI4K2A acts endogenously or exogenously depending on the tumor, or both, we do not know yet. It will require larger samples and adequate clinical studies to rectify our findings. In summary, we have shown that PI4K2A can act as an independent predictor of COAD, but our *results* require further validation in vivo or in vitro experiments.

## 5 CONCLUSIONS

In conclusion, we demonstrate that PI4K2A is a potent potential biomarker for early diagnosis, prognosis, and treatment of COAD. Signaling pathways including Chemokine signaling pathway, Hedgehog signaling pathway, MAPK signaling pathway, Pathways in cancer, and Toll‐like receptor signaling pathway may be the major regulatory pathways of PI4K2A. In addition, PI4K2A is an independent prognostic element of COAD and is closely interrelated with immunotherapy. Moreover, many anticancer drug sensitivities were negatively correlated with PI4K2A expression, providing some new ideas for the treatment of COAD. However, these findings of ours need to be further validated by in vivo and in vitro assays.

### AUTHOR CONTRIBUTION

Zhouyang Cheng and Bingye Zhu: Protocol/project development; Peng Bao: Data collection or management; Yang Cao: Data analysis; Xinkun Huang: Manuscript writing/editing.

## FUNDING INFORMATION

None declared.

## CONFLICT OF INTEREST

None declared.

## CONSENT FOR PUBLICATION

Not applicable.

## ETHICS APPROVAL AND CONSENT TO PARTICIPATE

This study was approved by the Institutional Research Ethics Committees of the Affiliated Hospital of Nantong University (NO.2022‐L130). We confirmed that all methods were carried out in accordance with relevant regulations and written informed consent was obtained from patients.

## Data Availability

All data were obtained from The Cancer Genome Atlas (TCGA) database. Please contact the corresponding author for data request.
